# Sialoendoscopy with Intraductal Irrigation in Chronic Salivary Gland Disease: A Minimally Invasive, Antibiotic-Sparing Strategy

**DOI:** 10.3390/antibiotics15040415

**Published:** 2026-04-20

**Authors:** Riccardo Manzella, Palmira Immordino, Francesco Lorusso, Francesco Dispenza, Federico Sireci, Cosimo Galletti, Salvatore Gallina, Angelo Immordino

**Affiliations:** 1 Otorhinolaringology Section, Department of Biomedicine, Neuroscience and Advanced Diagnostics, University of Palermo, 90127 Palermo, Italy; riccardo.manzella@community.unipa.it (R.M.); francesco.lorusso@policlinico.pa.it (F.L.); francesco.dispenza@unipa.it (F.D.); salvatore.gallina@unipa.it (S.G.); 2 Hygiene and Preventive Medicine Section, Department of Health Promotion, Maternal and Infant Care, Internal Medicine and Medical Specialties, University of Palermo, 90127 Palermo, Italy; palmira.immordino@unipa.it; 3 Otorhinolaryngology Section, Department of Precision Medicine in Medical, Surgical and Critical Care, University of Palermo, 90127 Palermo, Italy; federico.sireci@unipa.it; 4 Faculty of Medicine and Surgery, University of Enna “Kore”, 94100 Enna, Italy; cosimo.galletti01@unikore.it

**Keywords:** chronic sialadenitis, sialoendoscopy, intraductal irrigation, antibiotic stewardship, antibiotic-sparing therapy

## Abstract

**Background/Objectives**: Chronic and recurrent sialadenitis are inflammatory disorders of the major salivary glands often managed with repeated courses of systemic antibiotics, despite limited long-term efficacy and growing concerns regarding antimicrobial resistance. Minimally invasive intraductal therapies, including sialoendoscopy with irrigation, have emerged as effective alternatives aimed at addressing ductal obstruction and chronic inflammation while reducing antibiotic exposure. This study aimed to systematically review the available evidence on the effectiveness and safety of sialoendoscopy with intraductal irrigation in the management of chronic and recurrent sialadenitis, with particular attention to its potential antibiotic-sparing role. **Methods**: A literature review was conducted in accordance with PRISMA guidelines. Major scientific databases were searched to identify studies evaluating sialoendoscopy with intraductal irrigation in patients with chronic or recurrent sialadenitis. Study characteristics, patient populations, irrigation protocols, and clinical outcomes were extracted and qualitatively analyzed. **Results**: Sialoendoscopy with intraductal irrigation was associated with significant clinical improvement in more than two-thirds of patients, with complete or partial symptom resolution. The procedure demonstrated high technical feasibility and a favorable safety profile. Symptom control was maintained across most etiological subgroups. The need for prolonged or repeated systemic antibiotic treatment decreased following endoscopic intervention. **Conclusions**: Sialoendoscopy with intraductal irrigation may represent a promising and minimally invasive therapeutic option for chronic and recurrent sialadenitis and may contribute to improved antibiotic stewardship by reducing unnecessary systemic antibiotic use. These findings suggest that intraductal therapeutic strategies could be considered within evolving care pathways for chronic salivary gland disorders, aligning clinical management with broader public health efforts to combat antimicrobial resistance.

## 1. Introduction

Chronic sialadenitis is a persistent inflammatory disorder of the major salivary glands characterized by recurrent pain, swelling, and functional impairment, often exacerbated during salivary stimulation. It arises from a complex interplay of ductal obstruction, chronic inflammation, and salivary gland dysfunction, which may ultimately result in irreversible gland damage and functional loss if left untreated. Recurrent inflammatory episodes are frequently associated with repeated healthcare access and pharmacological interventions, contributing to a significant burden on patients’ quality of life. The etiology of chronic sialadenitis is multifactorial. Obstruction of the salivary ductal system by sialoliths, ductal stenoses, mucous plugs, or altered salivary composition leads to salivary stasis, recurrent infection, and chronic inflammation [1,2].

Autoimmune diseases, such as Sjögren’s syndrome [3], and iatrogenic factors, including radioactive iodine therapy for thyroid cancer [4] and post-radiotherapy changes, further compromise ductal patency and glandular function. Juvenile recurrent parotitis (JRP) represents a distinct inflammatory condition of unclear etiology, characterized by recurrent episodes of parotid gland swelling in children and adolescents, predominantly unilateral and only occasionally bilateral [5,6]. Although intraductal sialolithiasis remains the most common cause of obstruction [7], ductal stenoses account for approximately 15–25% of cases [8].

The natural history of chronic sialadenitis is marked by episodic exacerbations of pain, gland swelling, and salivary dysfunction, with a tendency toward progressive glandular atrophy. These recurrent flares are often treated empirically, commonly with repeated courses of systemic antibiotics even when clear evidence of acute bacterial infection is lacking. In this context, chronic sialadenitis represents a typical ENT condition where recurrent symptoms and diagnostic uncertainty may promote antibiotic overuse, raising concerns about treatment appropriateness and cumulative antibiotic exposure [9,10].

Historically, management strategies have ranged from conservative medical therapy to invasive surgical procedures, including gland excision. Conventional medical treatment includes hydration, sialogogues, gland massage, anti-inflammatory agents, and systemic antibiotics when bacterial infection is suspected. However, systemic antibiotics alone often fail to address the underlying ductal pathology or persistent intraglandular inflammation, particularly in chronic or recurrent cases. Moreover, repeated use of systemic antibiotics is increasingly recognized as a contributing factor to antimicrobial resistance (AMR) and treatment-related adverse effects, underscoring the need for more targeted therapeutic approaches in chronic ENT conditions [11,12]. In recent years, minimally invasive ductal therapies have significantly reshaped the management of chronic sialadenitis. Sialoendoscopy has emerged as a key diagnostic and therapeutic tool, allowing direct visualization of the ductal system, removal of obstructive material, dilation of stenoses, and intraductal irrigation [13,14]. This gland-preserving technique has demonstrated favorable outcomes in symptom control and functional recovery, while reducing the need for open surgery or gland excision [15,16,17]. Among intraductal therapeutic strategies, corticosteroid irrigation performed during sialoendoscopy has become widely adopted due to its ability to modulate chronic inflammation and reduce symptom recurrence. In addition, ductal irrigation with normal saline alone or combined with adjunctive agents such as steroids or antibiotics offers a less invasive option that has shown clinical benefit in selected populations, including patients with Sjögren’s syndrome and post-radiotherapy or post-radioiodine (RAI) sialadenitis. Within this context, intraductal antibiotic therapy has gained interest as a potential strategy to manage recurrent inflammatory episodes while limiting systemic antibiotic exposure. By delivering high local concentrations of antimicrobial agents directly to sites of ductal inflammation, this approach may improve therapeutic efficacy in selected cases and align with the principles of rational antibiotic use increasingly emphasized in ENT practice [18,19,20].

This study aimed to evaluate the effectiveness of interventional sialoendoscopy with intraductal irrigation in patients with recurrent sialadenitis, with particular attention to the clinical role and potential benefits of intraductal antibiotic therapy in the management of chronic and recurrent disease. From a public health perspective, recurrent ENT infections represent a significant contributor to inappropriate antibiotic prescribing, with implications for the growing threat of AMR. Recent evidence highlights the need for context-specific stewardship strategies in ENT practice, especially in high-prescribing settings where diagnostic uncertainty often leads to antibiotic overuse [21]. By delivering localized therapy directly to the affected ducts, intraductal antibiotic irrigation may reduce reliance on systemic antibiotics, supporting both individualized care and broader antimicrobial stewardship goals [21]. This study builds on this rationale by evaluating the potential of a targeted, minimally invasive approach to address chronic sialadenitis while advancing public health efforts against AMR [21,22].

## 2. Results

**Figure 1:**
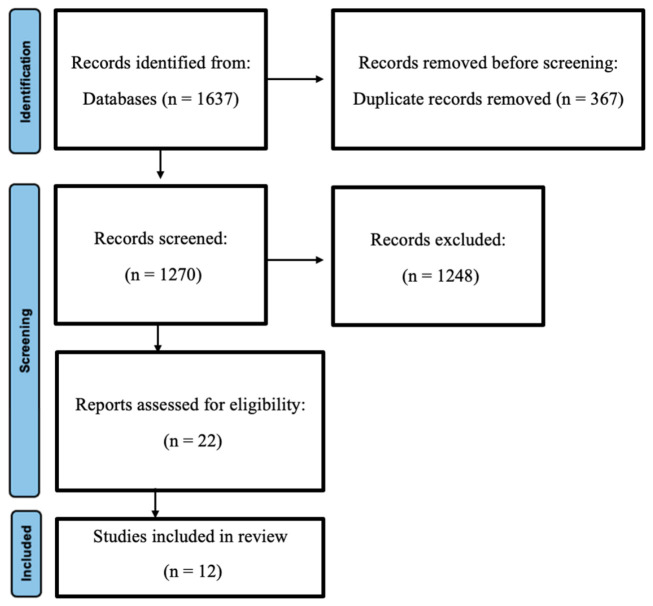
PRISMA 2020 flow diagram.

Our initial literature search yielded 1637 references. By applying the PRISMA 2020 flow diagram (Supplementary Materials), we excluded 367 duplicates, resulting in 1270 abstracts for review. Subsequently, 1248 articles were excluded by the research protocol criteria. The remaining 22 papers were then read in detail by two independent researchers. A discussion was held to reach a consensus on the inclusion or exclusion of each paper for the present study. Ultimately, only 12 papers met the eligibility criteria. Figure 1 shows the PRISMA 2020 flow diagram.

The twelve studies included a total of 397 patients, with a predominance of females (176 females vs. 104 males; sex distribution was not uniformly reported). The mean age ranged widely, including both pediatric and adult populations. 

From a topographic standpoint, the parotid gland was the most frequently involved, followed by the submandibular gland; bilateral involvement was less common and reported in three studies [23,24,25]. The main etiologies included chronic non-lithiasic obstructive sialadenitis, autoimmune disorders [23,26,27] (particularly Sjögren’s syndrome), post-actinic [26] or post-radioiodine injury [23,25,26], and idiopathic forms. In pediatric populations, juvenile recurrent parotitis was the predominant etiology [28,29,30]. Only two studies included patients with lithiasic sialadenitis [31,32].

Symptoms were reported in eight studies [23,25,26,28,29,31,32,33], with recurrent glandular swelling, pain, and xerostomia being the most common. One study reported symptom exacerbation during meals [23], and mucopurulent discharge was documented in only one study [30].

Diagnostic imaging was described in seven studies [24,26,28,29,30,33,34], with ultrasonography being the most commonly used modality [24,28,29,30,33,34]. Geisthoff et al. [28] identified a “leopard skin pattern” of multiple hypoechoic areas in all patients, whereas Berlucchi et al. [29] reported intraparenchymal lymphadenopathy, ductal dilatation, and microcalcifications. Additional imaging included sialography and salivary scintigraphy [26,33]. Table 1 shows the characteristics of the selected studies.

Five studies described conservative medical therapy prior to sialoendoscopy with intraductal irrigation [25,28,29,31,32], including systemic antibiotics, corticosteroids, NSAIDs, and sialogogues. While these therapies controlled acute exacerbations, they did not reduce the frequency or severity of episodes over one year, highlighting the potential for repeated antibiotic exposure in the absence of targeted therapy [9,10,11].

Sialoendoscopy with intraductal irrigation was evaluated across the included studies. Procedures were performed under local or general anesthesia, depending on patient characteristics. Irrigation protocols included single agents or combination regimens. Three studies used saline alone [25,28,33], three used corticosteroids exclusively (1 mL of 0.5% dexamethasone [26] or 2 mL of betamethasone, 4 mg/mL [24,27]), and combination protocols included corticosteroids with saline [23,29,30,31,32,33].

Irrigation was applied either as standalone therapy or after failure of conservative management. Sialoendoscopy was technically feasible in nearly all patients. Common intraoperative findings included mucous plugs, ductal stenosis, dense intraluminal material, ductal wall inflammation, intraductal debris, and pale mucosa [23,24,25,29].

The number of irrigation sessions varied: some studies performed a single session, while others repeated procedures for incomplete resolution [24,26]. Postoperative recommendations, including sialogogues and glandular massage, were reported in four studies [28,29,31,34] and were associated with reduced postoperative swelling.

Antibiotic use and its reporting across studies were heterogeneous and inconsistently described. To improve transparency, antibiotic-related outcomes were categorized as directly reported, indirectly inferred, or not reported (Table 2).

**Figure 2:**
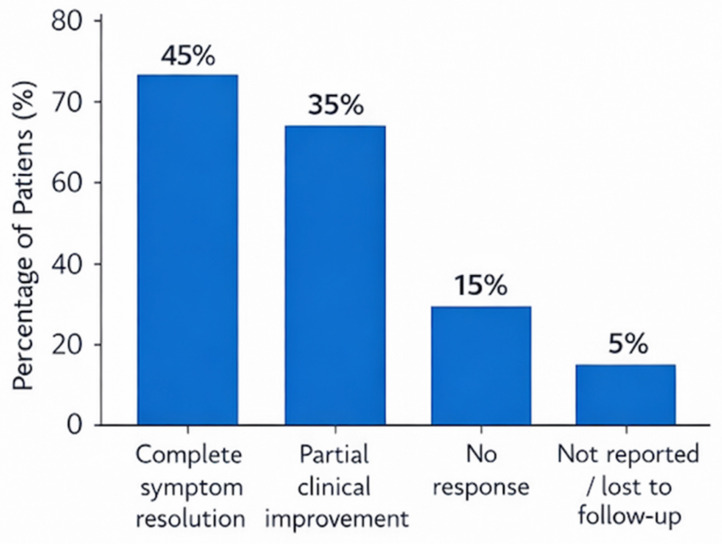
Clinical outcomes after sialoendoscopy with intraductal irrigation.

Clinical outcomes obtained are summarized in Figure 2.

The primary outcome—improvement of pain, swelling, and exacerbation frequency—was achieved in the majority of patients. Complete symptom resolution occurred in 35–55% of patients, while most others experienced partial remission, including reduced need for systemic antibiotics [9,10,11,33]. Lorusso et al. [31] observed additional improvement after repeated irrigation, with normalization of salivary flow and resolution of gland swelling. Overall, up to 80% of patients showed marked clinical improvement.

Only two studies [23,25] reported lack of response in single patients, likely due to irreversible glandular damage. Schwarz et al. [34] found no significant differences among irrigation regimens, supporting the mechanical rather than pharmacologic role of ductal clearance.

In pediatric patients with juvenile recurrent parotitis [28,29,30], ductal dilation and irrigation substantially reduced acute episodes, decreasing cumulative antibiotic exposure. Geisthoff et al. [28] reported two complete and four partial symptom resolutions among six patients; Berlucchi et al. [29] observed complete or partial remission in all cases.

Patients with post-radioiodine [23,25,26] or post-actinic [26] injuries showed variable improvement, with symptom improvement rates ranking from 60% to 90% [25,26]. Autoimmune sialadenitis, including Sjögren’s syndrome [23,26,27], showed comparable rates of symptom resolution. Capaccio et al. [27] reported an 87% reduction in swelling episodes with combined sialoendoscopy and intraductal corticosteroid irrigation, compared to 75% with sialoendoscopy alone.

Technical feasibility was high, with successful endoscopic access in nearly all patients. Complications were rare and minor, typically involving transient gland swelling. Importantly, follow-up confirmed sustained clinical benefit and a reduction in systemic antibiotic use across most patient subgroups, supporting sialoendoscopy as a minimally invasive, antibiotic-sparing intervention. To improve clarity and consistency in outcome reporting across the included studies, the primary and secondary outcomes considered in this review are defined in Table 3. Outcome interpretation is summarized in Table 4.

## 3. Discussion

Sialoendoscopy was initially introduced as a diagnostic and therapeutic modality for sialolithiasis; however, accumulating evidence has progressively supported the expansion of its indications beyond lithiasic disease. In recent years, this technique has been increasingly applied in the management of salivary duct stenosis and chronic inflammatory disorders of the major salivary glands [16]. Several studies have demonstrated that sialoendoscopy represents an effective and minimally invasive approach for both the diagnosis and treatment of non-lithiasic salivary gland obstruction and may be considered as a therapeutic option in selected patients, particularly after failure of conservative management [24,35,36,37,38].

From a therapeutic perspective, sialoendoscopy allows direct intraductal intervention through continuous lavage of the ductal system. This mechanism facilitates the mechanical removal of intraluminal debris, mucous plugs, and inflammatory material, thereby restoring ductal patency and improving salivary flow. The clearance of mucous plugs by saline irrigation appears to play a relevant role in symptom control and may contribute to reducing the risk of recurrent obstruction and persistent inflammation. These findings support the concept that chronic sialadenitis is frequently sustained by functional rather than fixed obstruction, making it particularly amenable to minimally invasive ductal interventions.

The present analysis confirms that sialoendoscopy combined with intraductal irrigation represents an effective and safe therapeutic option for the management of chronic non-lithiasic sialadenitis and recurrent inflammatory disorders of the major salivary glands. Across heterogeneous etiologies, more than two-thirds of patients experienced significant clinical benefit, achieving either complete or partial symptom resolution. These results reinforce the central role of gland-preserving, minimally invasive techniques in the contemporary management of chronic salivary gland disease.

The predominance of female patients and the frequent involvement of the parotid gland observed in the included studies are consistent with existing literature, particularly in autoimmune conditions and juvenile recurrent parotitis. The wide etiological spectrum highlights the multifactorial pathogenesis of chronic sialadenitis, in which ductal dysfunction, immune-mediated mechanisms, and iatrogenic injury interact to sustain chronic inflammation and symptom recurrence.

Subgroup analyses were not feasible due to the heterogeneity of the included studies and the limited availability of stratified data according to etiology. However, the underlying cause of sialadenitis may influence treatment outcomes, highlighting the need for future studies with stratified analyses.

Sialoendoscopy demonstrated high technical feasibility and a favorable safety profile across all included studies. The frequent identification of ductal stenosis, mucous plugs, and intraluminal debris supports the hypothesis that chronic functional obstruction plays a pivotal role in symptom persistence. Intraductal irrigation with saline and/or corticosteroids appears to reduce local inflammation, enhance ductal drainage, and augment the overall effectiveness of the endoscopic procedure.

The biological rationale for intraductal drug administration is supported by histopathological evidence showing that chronic sialadenitis is characterized by focal periductal inflammatory infiltrates predominantly composed of T lymphocytes [26]. Given the well-established anti-inflammatory and immunomodulatory effects of corticosteroids, particularly their ability to suppress T-cell-mediated immune responses, the use of intraductal corticosteroid irrigation in conjunction with sialoendoscopy may represent a valuable adjunctive strategy, potentially attenuating periductal inflammation and limiting the progression toward ductal fibrosis and irreversible glandular damage [39,40].

Regarding pharmacological efficacy, available evidence suggests that the clinical benefit of sialoendoscopy is primarily driven by mechanical ductal clearance rather than by the specific intraductal agent used. The heterogeneity of intraductal irrigation protocols across studies makes it difficult to distinguish the relative contribution of mechanical ductal intervention from that of specific pharmacological agents. In many cases, the observed clinical improvement may be primarily attributable to the mechanical effects of sialoendoscopy itself, including ductal dilation and clearance of obstructive material, rather than the specific intraductal solution used. Schwarz et al. [34], in a cohort of 94 patients treated for sialodochitis, reported no statistically significant differences in patient-reported outcomes among three irrigation protocols (normal saline, single-dose corticosteroid, and corticosteroid solution). These findings indicate that while intraductal pharmacological agents may contribute to symptom control, their incremental benefit over saline irrigation alone remains uncertain. Nevertheless, the selective use of intraductal antibiotics deserves consideration in specific clinical scenarios, such as recurrent inflammatory flares with suspected bacterial involvement or failure of conservative therapy. By delivering high local concentrations directly to the ductal system while minimizing systemic exposure, intraductal antibiotic therapy may represent a rational, targeted approach in carefully selected patients. Importantly, this strategy may help reduce repeated systemic antibiotic prescriptions, aligning with principles of rational antibiotic use increasingly emphasized in ENT practice [18,19].

### 3.1. Chronic Sialadenitis and Antibiotic Prescribing in ENT Practice

Chronic and recurrent sialadenitis represents a paradigmatic example of an ENT condition frequently associated with repeated empirical antibiotic prescriptions. In the absence of clear clinical or microbiological evidence of acute bacterial infection, patients often receive multiple courses of systemic antibiotics aimed at controlling inflammatory flares rather than addressing the underlying ductal pathology. This practice, while common, is supported by limited evidence and may contribute to cumulative antibiotic exposure, treatment-related adverse effects, and the development of antimicrobial resistance [9,10,11].

The findings of the present review highlight that symptom recurrence in chronic sialadenitis is primarily driven by ductal obstruction and persistent intraductal inflammation, rather than ongoing infection. Consequently, repeated systemic antibiotic therapy is unlikely to provide a durable benefit in the absence of targeted ductal intervention. These observations underscore the need for a shift from symptom-driven antibiotic prescribing toward mechanism-based treatment strategies in chronic salivary gland disorders.

### 3.2. Intraductal Therapy as an Antibiotic-Sparing Strategy

Within this framework, sialoendoscopy with intraductal irrigation may be conceptualized as an antibiotic-sparing intervention. By mechanically restoring ductal patency and modulating local inflammation, this approach directly targets the pathophysiological substrate of chronic sialadenitis, thereby reducing reliance on repeated systemic antibiotic courses. Several included studies reported a reduction in symptom frequency and severity following endoscopic treatment, which was often accompanied by a decreased need for subsequent antibiotic therapy, when reported.

However, it should be noted that only a limited number of included studies explicitly quantified antibiotic use before and after sialoendoscopy. In most cases, the reduction in systemic antibiotic therapy was indirectly inferred from decreased symptom recurrence or reported qualitatively without standardized metrics. Therefore, the potential antibiotic-sparing effect should be considered exploratory and hypothesis-generating rather than definitive, as it is supported by limited direct evidence and heterogeneous reporting across studies. Although the incremental clinical benefit of specific intraductal pharmacological agents over saline irrigation alone remains uncertain, the selective use of intraductal antibiotics may be justified in carefully selected patients. Importantly, local intraductal delivery allows high antimicrobial concentrations at the site of disease while limiting systemic exposure, aligning with contemporary antimicrobial stewardship principles [18,19].

### 3.3. Implications for Antimicrobial Stewardship and Health Equity

From a broader public health perspective, the integration of sialoendoscopy into the management pathway of chronic sialadenitis may contribute to more appropriate antibiotic use within ENT practice. Antimicrobial stewardship programs increasingly emphasize the importance of minimizing unnecessary systemic antibiotic exposure, particularly in chronic and recurrent conditions where alternative disease-modifying interventions are available [19,20].

Moreover, chronic sialadenitis often affects patients with autoimmune diseases, prior oncological treatments, or pediatric populations, who may experience repeated healthcare encounters and cumulative antibiotic exposure over time. Ensuring access to minimally invasive, gland-preserving treatments such as sialoendoscopy may therefore have implications for health equity, reducing long-term morbidity and dependence on systemic pharmacological therapies. However, access to sialoendoscopy remains variable across healthcare systems, highlighting the need for wider dissemination of expertise and resources to ensure equitable implementation of antibiotic-sparing strategies.

Favorable outcomes were consistently observed in pediatric patients with juvenile recurrent parotitis and in adults with chronic non-lithiasic sialadenitis, with marked and sustained reductions in recurrence rates and pain. In contrast, patients with post-radioiodine or post-actinic injury exhibited more variable responses, likely reflecting irreversible parenchymal damage and glandular fibrosis. Nonetheless, even in these subgroups, endoscopic treatment provided clinically meaningful symptom relief and reduced the need for prolonged systemic medical therapy or invasive surgical interventions.

The main limitations of this analysis include heterogeneity in study design, variability in irrigation protocols and outcome measures, and inconsistent reporting of long-term follow-up. Furthermore, the predominance of observational studies limits the strength of causal inferences. Despite these limitations, the consistency of favorable outcomes across independent cohorts supports the role of sialoendoscopy as a valuable therapeutic option within a stepwise management approach.

These findings also support broader calls for ENT-focused antibiotic-sparing interventions, as emphasized by recent efforts to integrate public health priorities into the management of antimicrobial resistance in upper airway infections [21,22].

Nevertheless, future prospective studies with standardized outcome measures are warranted to better define optimal intraductal irrigation protocols and clarify the specific indications for adjunctive intraductal antibiotic therapy within an antimicrobial stewardship framework.

## 4. Materials and Methods

This literature review was conducted in accordance with the Preferred Reporting Items for Systematic Reviews and Meta-Analyses (PRISMA) guidelines. The review protocol was prospectively registered in the PROSPERO database (International Prospective Register of Systematic Reviews; registration number CRD420261355999), and the methodology was predefined and consistently followed throughout all stages of the study.

A comprehensive search of the scientific literature was independently performed by two authors using the following electronic databases: PubMed (U.S. National Library of Medicine, Bethesda, MD, USA), MEDLINE (U.S. National Library of Medicine, Bethesda, MD, USA), EMBASE (Elsevier, Amsterdam, The Netherlands), Web of Science (Clarivate Analytics, Philadelphia, PA, USA), the Cochrane Library (Cochrane, London, UK), and Google Scholar (Google LLC, Mountain View, CA, USA).

The full search strategy was developed for each database using controlled vocabulary and free-text terms. The PubMed search string was as follows: ((“chronic sialadenitis”[Title/Abstract] OR “recurrent sialadenitis”[Title/Abstract] OR “obstructive sialadenitis”[Title/Abstract] OR “salivary gland inflammation”[Title/Abstract]) AND (“sialoendoscopy”[Title/Abstract] OR “intraductal irrigation”[Title/Abstract] OR “salivary duct irrigation”[Title/Abstract] OR “intraductal therapy”[Title/Abstract])) This strategy was adapted for other databases (EMBASE, Web of Science, Cochrane Library, and Google Scholar) using appropriate syntax.

Study selection was performed independently by two reviewers. After removal of duplicates, titles and abstracts were screened, followed by full-text assessment of potentially eligible studies. Discrepancies were resolved through discussion and consensus.

### 4.1. Eligibility Criteria and PICOTS Framework

Study selection was guided by the Population, Intervention, Comparison, Outcomes, Timing, and Setting (PICOTS) framework, as described below.

- *Population and inclusion criteria:* Studies including patients of any age (pediatric and adult populations), sex, and ethnicity diagnosed with chronic or recurrent sialadenitis were considered eligible. Included etiologies comprised chronic non-lithiasic obstructive sialadenitis, autoimmune-related sialadenitis (including Sjögren’s syndrome), post-radioiodine or post-actinic sialadenitis, juvenile recurrent parotitis, and idiopathic forms. Studies including patients with sialolithiasis were also considered eligible when intraductal irrigation was performed as part of the treatment strategy. Patients who had previously undergone conservative medical therapy, including systemic antibiotics or corticosteroids, were not excluded.
- *Intervention:* Eligible studies were required to report the use of sialoendoscopy with intraductal irrigation, either as a standalone procedure or following failure of conservative medical therapy. Intraductal irrigation protocols included: saline solution alone, corticosteroids alone, antibiotics alone, or combinations of saline solution with corticosteroids, antibiotics, and/or mucolytic agents. Both intraoperative and postoperative intraductal instillation protocols were considered.
- *Comparison:* Comparators varied across studies and included conservative medical therapy prior to sialoendoscopy, sialoendoscopy without intraductal pharmacological irrigation, and different intraductal irrigation agents or protocols. Due to the heterogeneity of study designs, the presence of a direct comparator group was not required for inclusion.
- *Outcomes:* The primary outcome was clinical improvement, defined as complete or partial resolution of symptoms, including recurrent gland swelling, pain, xerostomia, and frequency of inflammatory exacerbations. Secondary outcomes included the technical feasibility of sialoendoscopy, intraoperative findings, the need for repeated procedures, postoperative complications, recurrence rates, reduction in antibiotic use when reported, and long-term symptom control during follow-up. Reduction in antibiotic use was considered when explicitly reported or indirectly described by authors; however, no standardized metric for antibiotic consumption was consistently available across studies.
- *Timing:* Studies published from database inception to December 2025 were included, with no lower date limit applied. No minimum follow-up duration was required; however, when available, both short- and long-term outcomes were extracted and analyzed.
- *Setting:* Eligible study designs included randomized controlled trials (RCTs), non-randomized controlled trials (NRCTs), prospective and retrospective cohort studies, and case–control studies conducted in community hospitals, private practice settings, and tertiary referral university hospitals. Case reports and small case series were excluded, unless they provided relevant data on intraductal antibiotic irrigation protocols.

### 4.2. Data Extraction and Synthesis

Data extracted from each included study encompassed study design, sample size, patient demographics, gland involvement, etiology, imaging findings, type and number of sialoendoscopic procedures, intraductal irrigation protocols, outcomes, complications, and follow-up duration. Due to substantial heterogeneity across studies in terms of study design, patient populations, underlying etiologies, intervention protocols, and outcome measures, a quantitative meta-analysis was not considered appropriate. Reported outcome percentages were derived from the ranges described in the included studies and should be interpreted as approximate values rather than weighted pooled estimates. Therefore, a qualitative synthesis was performed.

### 4.3. Risk of Bias Assessment

A formal risk of bias assessment was conducted at the study level. Given the predominance of observational studies and the heterogeneity of study designs, methodological quality was assessed qualitatively using domains adapted from established tools, including the Newcastle–Ottawa Scale (NOS) for observational studies and the Cochrane RoB 2 framework for randomized studies. The assessment focused on key domains, including selection of participants, comparability of study groups, and outcome assessment. Each study was evaluated and classified as having low, moderate, or high risk of bias across these domains.

Overall, the included studies showed a moderate to high risk of bias. The main sources of bias included retrospective study design, small sample sizes, lack of control groups, and heterogeneity in outcome definitions and follow-up duration. A summary of the risk of bias assessment is provided in Table 5.

### 4.4. Certainty of Evidence (GRADE)

The certainty of evidence was assessed using the Grading of Recommendations Assessment, Development and Evaluation (GRADE) approach.

Overall, the certainty of evidence was rated as low to very low. This rating was primarily driven by the observational nature of most included studies, which start at low certainty according to GRADE criteria. Further downgrading was applied due to risk of bias, inconsistency across studies, heterogeneity in patient populations and interventions, and imprecision related to small sample sizes.

No upgrading factors (such as large effect size or dose–response relationship) were consistently identified across studies. Therefore, while the direction of effect appears favorable, confidence in the magnitude of the effect remains limited.

These findings highlight the need for well-designed prospective and randomized studies to strengthen the evidence base.

## 5. Conclusions

Chronic and recurrent Sialadenitis poses a persistent therapeutic challenge, frequently resulting in repeated empirical antibiotic prescriptions despite limited efficacy in non-acute and obstructive–inflammatory disease. In the current era of escalating antimicrobial resistance, ENT conditions represent a critical frontline for implementing antibiotic stewardship strategies [9,10,11,18,19].

The findings of this study support that sialoendoscopy with intraductal irrigation may represent a promising and minimally invasive therapeutic option, particularly in patients who do not respond to conservative management.

By directly addressing ductal obstruction and localized inflammation, this minimally invasive approach provides durable symptom relief while preserving glandular function. Importantly, it offers a therapeutic alternative that may reduce reliance on prolonged or repeated systemic antibiotic therapy, which is often prescribed in the absence of clear microbiological evidence [9,10,11,18,19].

From a public health perspective, the adoption of intraductal, locally targeted therapies aligns with key principles of antimicrobial stewardship, including diagnostic precision, minimization of systemic antibiotic exposure, and optimization of treatment effectiveness [18,19]. This is particularly relevant in chronic ENT conditions, where inappropriate or excessive antibiotic use contributes disproportionately to the development of antimicrobial resistance and exposes patients to avoidable adverse effects.

Although outcomes remain variable among patients with irreversible parenchymal damage, such as post-radioiodine or post-radiotherapy siaoladenitis, sialoendoscopy remains a valuable symptom-modifying intervention that can reduce the need for invasive surgery or long-term medical therapy.

However, given the predominance of small, retrospective, and single-center studies, these findings should be interpreted with caution. Higher-quality prospective and randomized studies with standardized outcome measures and long-term follow-up are needed to confirm these results and better define the role of sialoendoscopy in clinical practice. Future studies should also incorporate standardized quantitative metrics, such as the number of antibiotic prescriptions per patient-year or defined daily doses before and after intervention.

In conclusion, sialoendoscopy with intraductal irrigation represents not only an effective clinical tool but also a strategy with meaningful public health implications. Its integration into routine care pathways for chronic sialadenitis may help reconcile optimal patient-centered care with the global imperative to preserve antibiotic efficacy [9,10,11,18,19].

## Figures and Tables

**Table 1:** Characteristics of the selected studies.

Author	Year	Study Design	Patients (*n*)	Etiology	Intraductal Irrigation Protocol
Antoniades et al. [32]	2004	Retrospective	82	Chronic sialadenitis	Penicillin with saline vs. saline
Prendes et al. [25]	2012	Retrospective	11	Post-radioiodine sialadenitis	Saline
Schneider et al. [30]	2014	Retrospective	15	Juvenile recurrent parotitis	Saline + steroid irrigation
Capaccio et al. [24]	2017	Prospective	18	Recurrent idiopathic sialadenitis	Steroid irrigation
Capaccio et al. [27]	2018	Pilot monocentric trial	12	Sjögren’s syndrome	Steroid irrigation
Berlucchi et al. [29]	2018	Retrospective	23	Juvenile recurrent parotitis	Saline + steroid irrigation
Schwarz et al. [34]	2018	Retrospective	94	Sialadenitis without sialolithiasis	Saline vs steroid irrigation vs. Saline + steroid irrigation
Lele et al. [23]	2019	Retrospective	33	Chronic sialadenitis, autoimmune sialadenitis, post-radioiodine sialadenitis, idiopathic	Saline + steroid irrigation
Kim et al. [33]	2020	Prospective	33	Chronic obstructive sialadenitis	Saline irrigation
Chen et al. [26]	2021	Retrospective	28	Sjögren’s syndrome, Post-radioiodine/actinic sialadenitis	Steroid irrigation
Geisthoff et al. [28]	2022	Retrospective	6	Juvenile recurrent parotitis	Saline irrigation
Lorusso et al. [31]	2022	Prospective	26	Chronic obstructive sialadenitis	Mucolytic + steroid + antibiotic

**Table 2:** Reporting of antibiotic use across included studies.

Author	Antibiotic Use Reported	Type of Reporting
Antoniades et al. [32]	Yes	Direct
Prendes et al. [25]	No	Not reported
Schneider et al. [30]	No	Not reported
Capaccio et al. [24]	No	Indirect
Capaccio et al. [27]	No	Indirect
Berlucchi et al. [29]	No	Indirect
Schwarz et al. [34]	No	Not reported
Lele et al. [23]	No	Indirect
Kim et al. [33]	No	Not reported
Chen et al. [26]	No	Indirect
Geisthoff et al. [28]	No	Indirect
Lorusso et al. [31]	Yes	Direct

**Table 3:** Definition of primary and secondary outcomes.

Outcome	Definition
Complete resolution	Absence of symptoms after treatment
Partial improvement	Reduction in symptom frequency or severity
Recurrence reduction	Decrease in number of episodes over time
Antibiotic use reduction	Reported decrease in systemic antibiotic prescriptions

**Table 4:** Outcome interpretation after sialoendoscopy with intraductal irrigation.

Clinical Outcome	Reported Rate	Interpretation
Complete symptom resolution	35–55%	Full remission
Partial clinical improvement	≈45%	Reduced pain, swelling, and recurrence
Overall clinical improvement	Up to 80%	Complete + partial response
Lack of response	<5%	Likely irreversible gland damage
Technical success	>95%	High feasibility
Complications	Rare, minor	Transient swelling

**Table 5:** Risk of bias assessment of included studies.

Author	Study Design	Selection	Comparability	Outcome	Overall Risk of Bias
Antoniades et al. [32]	Randomized Controlled Trial	Low	Moderate	Moderate	Moderate
Prendes et al. [25]	Observational	Moderate	Low	Moderate	Moderate
Schneider et al. [30]	Observational	Moderate	Low	Moderate	Moderate
Capaccio et al. [24]	Observational	Moderate	Moderate	Moderate	Moderate
Capaccio et al. [27]	Observational	Moderate	Moderate	Moderate	Moderate
Berlucchi et al. [29]	Observational	Moderate	Low	Moderate	Moderate
Schwarz et al. [34]	Observational	Moderate	Low	Moderate	Moderate
Lele et al. [23]	Observational	Moderate	Low	Moderate	Moderate
Kim et al. [33]	Observational	Moderate	Low	Moderate	Moderate
Chen et al. [26]	Observational	Moderate	Low	Moderate	Moderate
Geisthoff et al. [28]	Observational	Moderate	Low	Moderate	Moderate
Lorusso et al. [31]	Observational	Moderate	Moderate	Moderate	Moderate

## Data Availability

No new data were created or analyzed in this study. Data sharing is not applicable to this article.

## Supplementary Materials

The following supporting information can be downloaded at: https://www.mdpi.com/article/10.3390/antibiotics15040415/s1, PRISMA 2020 Checklist [41].

## Abbreviations

The following abbreviations are used in this manuscript:

PRISMA	Preferred Reporting Items for Systematic Reviews and Meta-Analyses
ENT	Ear, Nose and Throat
JRP	Juvenile recurrent parotitis
AMR	Antimicrobial resistance
RAI	Post-radioiodine
